# Identification and characterization of human ovary-derived circular RNAs and their potential roles in ovarian aging

**DOI:** 10.18632/aging.101565

**Published:** 2018-09-23

**Authors:** Hongcai Cai, Yamin Li, Huimin Li, Jean Damascene Niringiyumukiza, Mengdi Zhang, Li Chen, Gang Chen, Wenpei Xiang

**Affiliations:** 1 Family Planning Research Institute/Center of Reproductive Medicine, Tongji Medical College, Huazhong University of Science and Technology, Wuhan, Hubei 430030, China; 2 Department of Obstetrics and Gynecology, Tongji Hospital, Tongji Medical College, Huazhong University of Science and Technology, Wuhan, Hubei 430030, China; * Equal contribution

**Keywords:** human, ovary, circRNA, aging, senescence, granulosa cell

## Abstract

Circular RNAs (circRNAs) have recently been shown to exert effects on multiple pathological processes by acting as miRNA sponges. However, the roles of circRNAs in ovarian senescence are largely unknown. The objective of this study was to identify the circRNAs involved in ovarian aging and predict their potential biological functions. We first performed RNA-sequencing to generate ovarian circRNA expression profiles from young (n = 3) and aging (n = 3) groups. In total, 48,220 circRNAs were identified, of which 194 circRNAs were significantly up-regulated and 207 circRNAs were down-regulated during aging (fold change > 2, *P* < 0.05). Bioinformatics analysis demonstrated that the metabolic process, regulated secretory pathway, oxidation-reduction process, steroid hormone biosynthesis, and insulin secretion pathways, which may be associated with ovarian aging, were significantly enriched (*P* < 0.05). The biological characteristics of ovary-derived circRNA, such as back-splicing, RNase R resistance, stability, and alternative splicing, were further validated. Bioinformatics predicted that most of the circRNAs harboured miRNA binding sites, of which *circDDX10*-*miR-1301-3p/miR-4660*-*SIRT3* axis may be involved in the regulation of ovarian function. Our study indicates that circRNAs are aberrantly expressed in the aging ovary and may play potential roles in the development of ovarian senescence.

## Introduction

Ovarian senescence is an age-related decline of female reproductive capacity, characterized by a gradual reduction in the quantity and quality of oocytes [1,2]. Neuroendocrine, genetic and environmental factors are involved in the regulation of ovarian senescence [3]. Poor oocyte quality, including meiotic abnormalities, mitochondrial defects, and ooplasm quality, are clearly linked to adverse reproductive outcomes [4]. Given the role of the epigenome in controlling gene expression and chromatin structure, it is likely a contributor to the decline in fecundity in aging females [5]. Non-coding RNAs (ncRNAs), such as miRNAs, piRNAs, and lncRNAs, are important regulators of gene expression, particularly at the post transcriptional level, and are important members of the epigenetic regulation network [6]. In recent years, a new type of ncRNA, circular RNA (circRNA), has attracted attention. It is a rising star of the RNA family after the discovery of miRNAs and lncRNAs, and it is also the latest research hotspot in the RNA family [7].

CircRNAs were once considered splicing by-products or artefacts until the recent development in RNA-sequencing (RNA-seq) technology [8]. Unlike traditional linear RNAs, circRNA forms a covalently closed continuous loop, which is resistant to nucleic acid exonuclease [9]. During the past decades, circRNAs were identified in various eukaryotic tissues and cells from *Drosophila*, nematodes, mice, monkeys, cows and humans, and presented significant tissue- and developmental stage-specificity [10–13]. The intron circRNA (ciRNA) and exon intron circRNA (EIciRNA) located in the nucleus function as *cis* regulators of host gene expression, whereas the circRNA located in the cytoplasm is a competitive endogenous RNA (ceRNA) and becomes miRNA "sponge", thus regulating expression of the corresponding target gene [14]. More recently, several studies have found that circRNAs could encode proteins *in vivo* and may participate in important biological activities [15,16].

In the past few years, various studies reported the role and mechanism of circRNA in cardiovascular diseases [17], diabetes [18], tumours [19] and nervous system diseases [20]. CircRNA may be a potential biomarker for the diagnosis and prognosis of many diseases, and its dysregulated expression may play an important role in the pathogenesis of many human diseases [21,22]. More recently, circRNA was reported to be closely associated with cell senescence and cell survival. In studies of fruit flies [12], monkeys [11], mice [10] and nematodes [13], researchers discovered a large number of circRNAs closely related to cell senescence, which might be used as "miRNA sponges" to promote the expression of downstream target genes by binding to the corresponding miRNAs, thereby participating in the occurrence and development of cell senescence and senescence-related diseases. In 2014, researchers first identified the expression profile of circRNAs in *Drosophila* ovarian tissue [23]. Subsequently, the potential effects of circRNAs in pre-ovulatory ovarian follicles of goats were discovered [24]. More recently, Chen and colleagues revealed the potential effects of circRNAs on ovary activation and oviposition in honey bees [25]. Given the previous studies in reproductive processes, the investigation of circRNAs in human ovaries would provide a valuable opportunity to understand the molecular basis of human reproduction.

Therefore, to further explore the key factors in the regulation of ovarian function and better understand the complicated regulatory network of ovarian aging, high-throughput sequencing and bioinformatics analyses of circRNAs in normal human ovaries were performed. Tens of thousands of novel circRNAs were identified for the first time in the human ovary. The general molecular biological characteristics of circRNAs were also determined. A circRNA-miRNA-mRNA network related to ovarian senescence was subsequently constructed to explore the interaction among different molecules.

## RESULTS

### Overview of circRNA expression in human ovarian tissues

**Figure 1:**
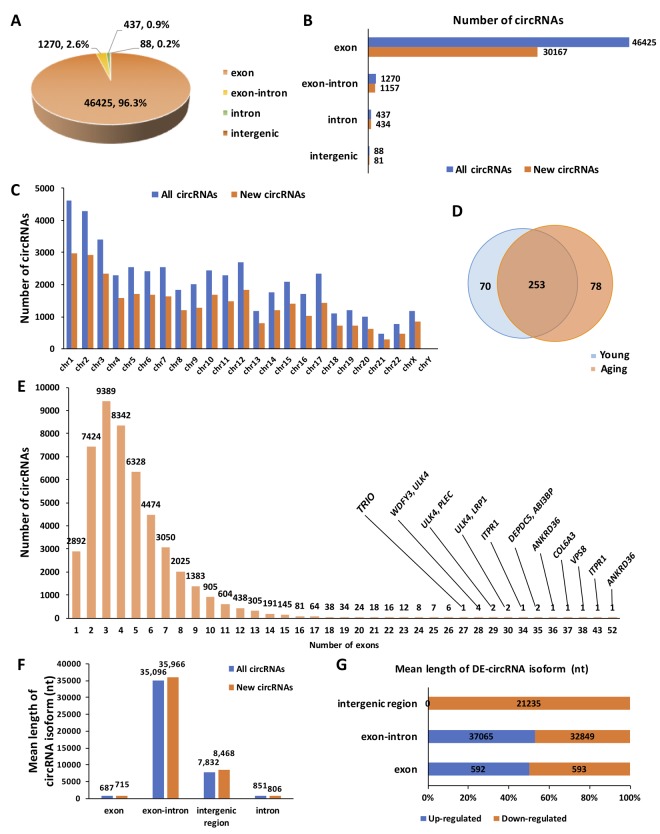
**Overview of circRNA expression in human ovarian tissues.** (**A**) The proportion of different types of circRNAs among all predicted circRNAs. (**B**) The proportion of newly discovered circRNAs among different types of circRNAs. (**C**) The distribution of newly discovered circRNAs in the human genome. (**D**) Differentially expressed (DE) circRNAs between young and aging human ovarian tissues. (**E**) The distribution of exons among all predicted circRNAs. (**F**) The mean length of circRNA isoforms among all predicted and newly discovered circRNAs. (**G**). The mean length of DE-circRNA isoforms.

A total of 48,220 circRNAs were identified in the ovary tissues, of which 31,839 were novel based on comparison with other databases (e.g., circBASE, CIRCpedia). The majority of circRNAs were exonic (96.3%), whereas only a small proportion of circRNAs contained introns and unannotated intergenic regions (Fig. 1A). The distribution of newly discovered circRNAs among different circRNA isoforms was presented in Fig. 1B. Commonly, circRNAs were widely distributed across all chromosomes. Chromosome 1 and chromosome 2 both produced over 4,000 circRNAs, whereas most of the other chromosomes generated 1,000–3,000 circRNAs. The distribution of different types of circRNAs in the human genome was shown in Fig. 1C. There were 70 circRNAs specifically expressed in the young group, 78 circRNAs specifically expressed in the aging group, and 253 circRNAs commonly expressed in both groups (Fig. 1D). For exonic circRNA, there were up to 52 exons in one single circRNA (*circANKRD36*). However, we found that the majority (25,155, > 54%) of exon circRNAs were composed of 2 to 4 exons within the same host genes (Fig. 1E). The mean lengths of exonic circRNAs were 687 and 715 nucleotide (nt) in the total and newly discovered circRNAs, respectively. Additionally, the mean lengths of other circRNA isoforms were also demonstrated in Fig. 1F. The mean lengths of circRNA isoforms for up-regulated and down-regulated circRNAs were similar (Fig. 1G). Details for all the circRNAs identified in this study are provided in Supplementary Table S1.

### Identification and functional annotation of differentially expressed (DE) circRNAs

**Figure 2:**
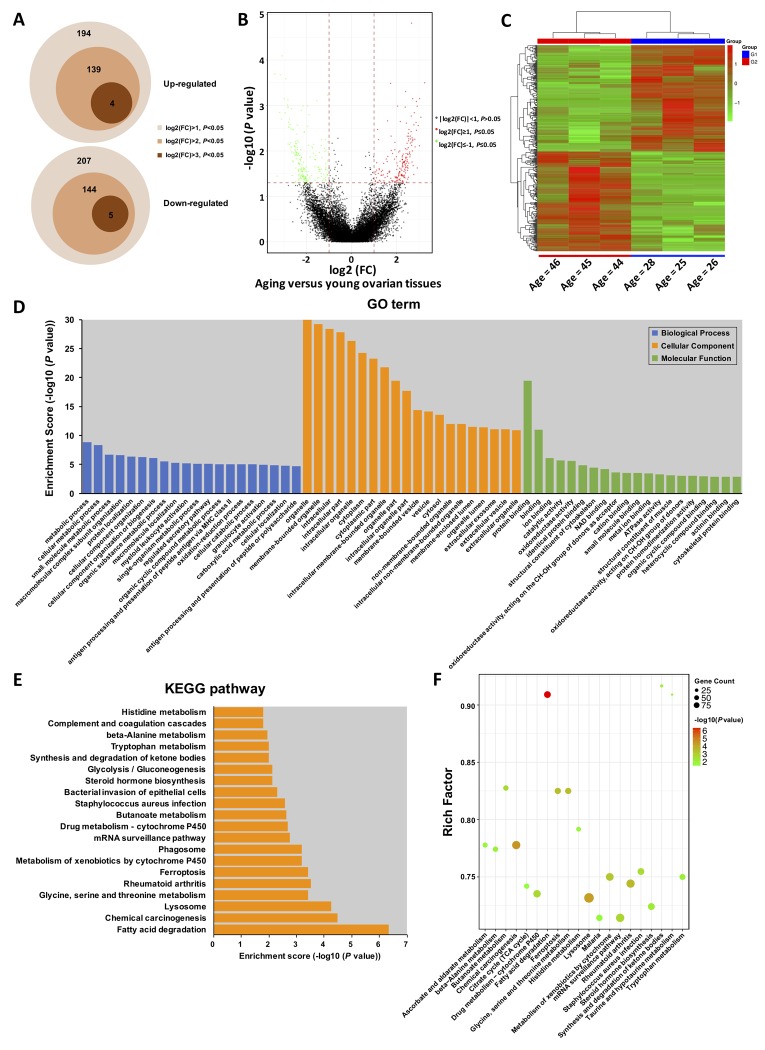
**Gene Ontology (GO) and Kyoto Encyclopedia of Genes and Genomes (KEGG) pathway annotation of host genes.** (**A**) Schematic diagram of the DE-circRNAs between young and aging groups. (**B**) The volcano plot is constructed using fold-change and P-values. The vertical lines correspond to 2.0-fold up- and down-regulation between YA and AA samples, and the horizontal line represents a *P*-value. Green and red circles represent differentially expressed (DE) circRNAs with statistical significance (fold change > 2.0 and *P* < 0.05). Green circles represent the down-regulated circRNAs, whereas red circles represent the up-regulated circRNAs. (**C**) Hierarchical clustering analysis of DE-circRNAs in the ovarian tissues of three young (< 30 years) and three older (> 40 years) women. The expression values (Fold change > 2.0, *P* < 0.05) were represented in different colours, indicating expression levels above and below the median expression level across all samples. (**D**) Top 20 GO terms in each category, and top 20 KEGG signalling pathway annotations (**E** and **F**).

Circular RNAs are often expressed in the tissue and are developmental stage specific. In particular, during ovarian aging, circRNA expression levels exhibit dynamic global changes. DEGseq analysis (fold change > 2, *P* < 0.05) identified a total of 401 DE-circRNAs (fold change > 2, *P* < 0.05) in the young (n = 3) and aging (n = 3) groups, consisting of 194 up-regulated and 207 down-regulated circRNAs. Of them, 139 circRNAs were four folds significantly up-regulated, and four circRNAs were eight folds significantly up-regulated. Moreover, 144 circRNAs were four folds significantly down-regulated, and five were eight folds significantly down-regulated (Fig. 2A). The volcano plot and heat map indicated that the DE-circRNAs were clustered based on their expression profiles (Fig. 2B, C). Furthermore, the distribution and characteristics of the DE-circRNAs were exhibited in Supplementary Fig. S1. Details of the DE-circRNAs identified in this study were presented in Supplementary Table S2.

### *Gene Ontology (GO) and Kyoto Encyclopedia of Genes and Genomes (KEGG) enrichment of DE-circRNAs*

One thousand one hundred and twenty-three GO terms were significantly enriched (FDR threshold of < 0.05) for the host genes of the DE-circRNAs. As shown in Fig. 2D, the top 20 significantly enriched biological processes, cellular components, and molecular functions were metabolic process, regulated secretory pathway, oxidation-reduction process, organelle, vesicle, extracellular exosome, protein binding, ion binding, catalytic activity, and oxidoreductase activity, most of which were mainly associated with the process of aging. Additionally, the GO terms for the targeted genes of the DE-circRNAs were also presented in Supplementary Fig. S2. Details of the GO enrichment with statistical significance were presented in Supplementary Table S3. Forty-six KEGG pathways (FDR *P* value < 0.05) were associated with the DE-circRNAs. As shown in Fig. 2E, F, the top 20 enriched pathways were highly associated with fatty acid degradation, lysosomes, glycine, serine and threonine metabolism, steroid hormone biosynthesis, glycolysis/gluconeogenesis, synthesis and degradation of ketone bodies, and other important amino acid metabolism processes. Similarly, we further performed KEGG pathway analysis for the targeted genes of the DE-circRNAs, which were shown in Supplementary Fig. S3. Details of the KEGG enrichment with statistical significance were presented in Supplementary Table S4.

### Experimental validation of circRNA-sequencing

**Figure 3:**
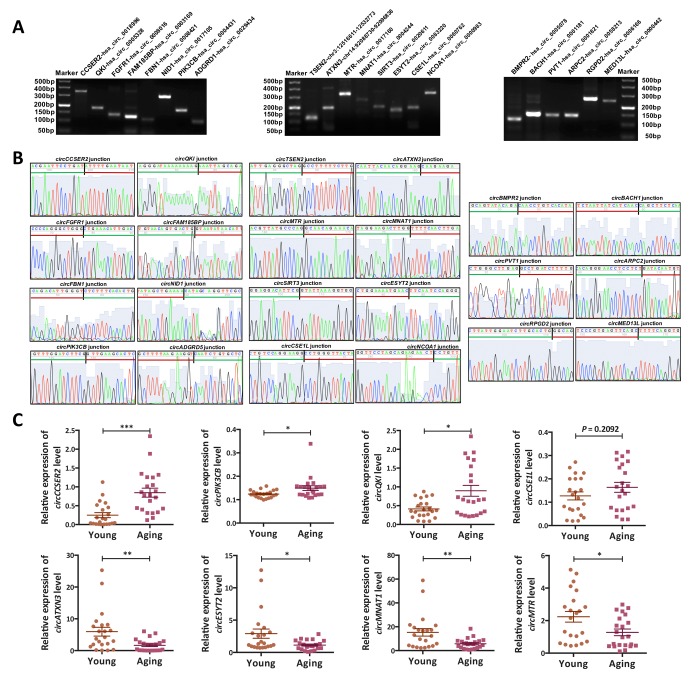
**Validation of the circRNA-sequencing results.** (**A**) PCR products (eight up-regulated, eight down-regulated and six commonly expressed circRNAs) were examined by 2% agarose electrophoresis. (**B**) The back-spliced junction of the randomly selected 22 circRNAs was confirmed by sanger sequencing. The black vertical line represented the back-spliced junction sites. The green horizontal line indicated the 5' end and the red line indicated the 3' end of the circRNA sequence. (**C**) The expression levels of eight differentially expressed (DE) circRNAs in a larger cohort (n = 44) were examined by qPCR. *, *P* < 0.05; **, *P* < 0.01; ***, *P* < 0.001.

Several circRNAs (*circCCSER2*, *circPIK3CB*, *circQKI*, *circCSE1L*, *circATXN3*, *circESYT2*, *circMNAT1* and *circMTR*) identified as DE-circRNAs by high-throughput sequencing were randomly selected for validation (Fig. 3). The qRT-PCR was performed to detect the expression of these circRNAs in young (n = 22) and aging (n = 22) ovarian cortex samples, including those used for RNA-seq. Agarose gel electrophoresis and Sanger sequencing were used to verify the specificity of the qRT-PCR products (Fig. 3A, B). Melting curve analysis of each circRNA validated was presented in Supplementary Fig. S4. As shown in Fig. 3C, the results of the qRT-PCR in a larger cohort (n = 44) were highly consistent with the RNA-seq results.

### Biological characteristics of these ovary derived circRNAs

### *Circularization feature*

**Figure 4:**
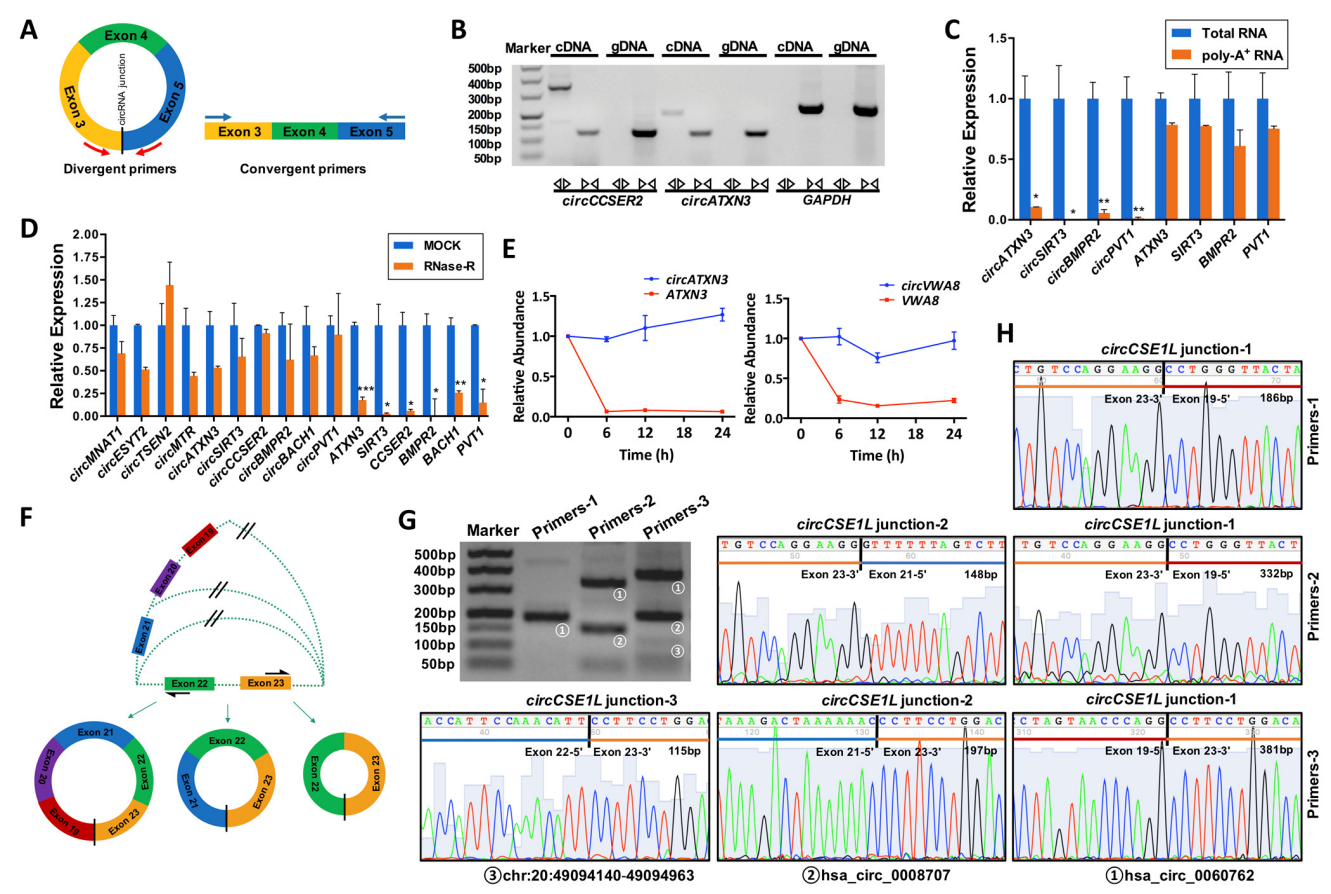
**General biological properties of ovary-derived circRNAs.** (**A**) Primer patterns of circRNAs and linear RNAs. The blocks represent exons. Red arrows represent divergent primers, blue arrows represent convergent primers, and the black vertical line represents the circRNA back-spliced junction. (**B**) CircRNAs were successfully amplified by divergent primers from cDNA but could not be amplified from gDNA. Linear RNAs were successfully amplified by convergent primers from both cDNA and gDNA. PCR products were examined by 2% agarose electrophoresis. The *circCCSER2* and *circATXN3* were used as experimental groups, and *GAPDH* was the control group. (**C**) Total RNA derived cDNA successfully amplified circRNAs, but this did not work for poly-A^+^ RNA derived cDNAs. Four circRNAs were examined, and their linear forms were used as controls. (**D**) CircRNAs were resistant to RNase R digestion, whereas their linear forms were sensitive to RNase R digestion. Ten circRNAs were examined, and six of the corresponding linear RNAs were used as controls. (**E**) Ovary-derived circRNAs can be stably expressed in human granulosa cells (hGCs) from follicular fluid in 24 h, whereas their linear forms are degraded rapidly. (**F**) A schematic diagram of the alternative splicing. PCR products for three pairs of primers were examined by 2% agarose electrophoresis (**G**) and Sanger sequencing (**H**). *CircCSE1L* was explored as an example. *, *P* < 0.05; **, *P* < 0.01; ***, *P* < 0.001.

Unlike linear RNAs, circRNAs are mainly formed by the upstream 3' end and the downstream 5' end reverse splicing form, and the circRNA ring diagram is shown below (Fig. 4A). Using divergent primers, circRNAs were successfully amplified from cDNA other than gDNA. However, unlike circRNAs, linear RNAs were successfully amplified by convergent primers from both cDNA and gDNA. PCR products were confirmed by 2% agarose electrophoresis. The *circCCSER2* and *circATXN3* were used as experimental groups, and *GAPDH* was the control group (Fig. 4B). Total RNA and polyA^+^ RNA derived cDNA were used to amplify several circRNAs (*circATXN3*, *circSIRT3*, *circBMPR2* and *circPVT1*) and their linear forms using divergent and convergent primers, respectively. As shown in Fig. 4C, total RNA and polyA^+^ RNA derived cDNA amplified linear RNAs, whereas polyA^+^ RNA derived cDNA could not amplify circRNAs, indicating their special circular structures.

### *RNase R resistance*

We randomly selected ten circRNAs (*circMNA1*, *circESYT2*, *circMTR*, *circATXN3*, *circSIRT3*, *circCCSER2*, *circBMPR2*, *circBACH1*, *circPVT1*) and six corresponding linear RNAs (*ATXN3*, *SIRT3*, *CCSER2*, *BMPR2*, *BACH1*, *PVT1*) as controls for the RNase R resistance study. The results of qRT-PCR revealed that the expression of all linear RNAs significantly decreased after 15 min of RNase R digestion (*P* < 0.05); however, most of the circRNAs were only slightly reduced (*P* > 0.05). This indicated that circRNAs were resistant to RNase R digestion, whereas linear RNA was sensitive to RNase R treatment (Fig. 4D).

### *Ovary-derived circRNAs are stably detected in granulosa cells (GC) from follicular fluid*

GCs were harvested and purified by Percoll density gradient centrifugation and further identified by immunofluorescence. Immunofluorescence revealed that over 95% of cells were marked with FSHR fluorescence in the samples (Supplementary Fig. S5). To address the stability, we detected the expression of 4 pairs of circRNAs (*circATXN3*, *circVWA8*, *circDDX10*, and *circPIK3CB*) and their linear RNAs (*ATXN3*, *VWA8*, *DDX10* and *PIK3CB*) in GCs at different time points. The results showed that circRNAs were more stable than their linear RNAs at 24 h after isolation from the follicular fluid (Fig. 4E and Supplementary Fig. S6).

### *Alternative splicing*

One gene can generate several different circRNAs, which consist of different exons and/or introns. We selected three circRNAs (hsa_circ_0060762, hsa_circ_0008707 and chr20:49094140-49094963) generated by the same gene, *CSE1L*, for alternative splicing analysis. Divergent primers for these circRNAs were designed at different exons as illustrated in Fig. 4F. Sequences of the primers and lengths of the PCR products were listed in Table 1. Primers-1 (located on exon 19 and exon 23) only amplified hsa_circ_0060762, primers-2 (located on exon 21 and exon 23) amplified both hsa_circ_0060762 and hsa_circ_0008707, and primers-2 (located on exon 22 and exon 23) amplified all three circRNAs, which were confirmed by electrophoresis and Sanger sequencing of PCR products (Fig. 4G, H).

**Table 1:** Primer sequences and aim products of circRNAs for alternative splicing analysis.

**Primers**	**Primer sequences**	**Exon composition**	**Aim products**	**Product length (bp)**
*circCSE1L-1* (Primers-1)	F: AGAGCATGATCCTGTAGGT R: GGCATGTGCTCTATTATACTG	23-19	hsa_circ_0060762	186
*circCSE1L-2* (Primers-2)	F: AGAGCATGATCCTGTAGGT R: CTTGTAGTGCTAGTGCCCCAT	23-19	hsa_circ_0060762	332
		23-21	hsa_circ_0008707	148
*circCSE1L-3* (Primers-3)	F: CAAGTTGTCTACCGCCTGTC F: CCAACCGCACAGATCTTTTTCT	23-19	hsa_circ_0060762	388
		23-21	hsa_circ_0008707	197
		23-22	chr20:49094140-49094963	115

F, forward; R, reverse.

### Prediction of ovarian aging-associated circRNA-miRNA-mRNA interaction network

**Figure 5:**
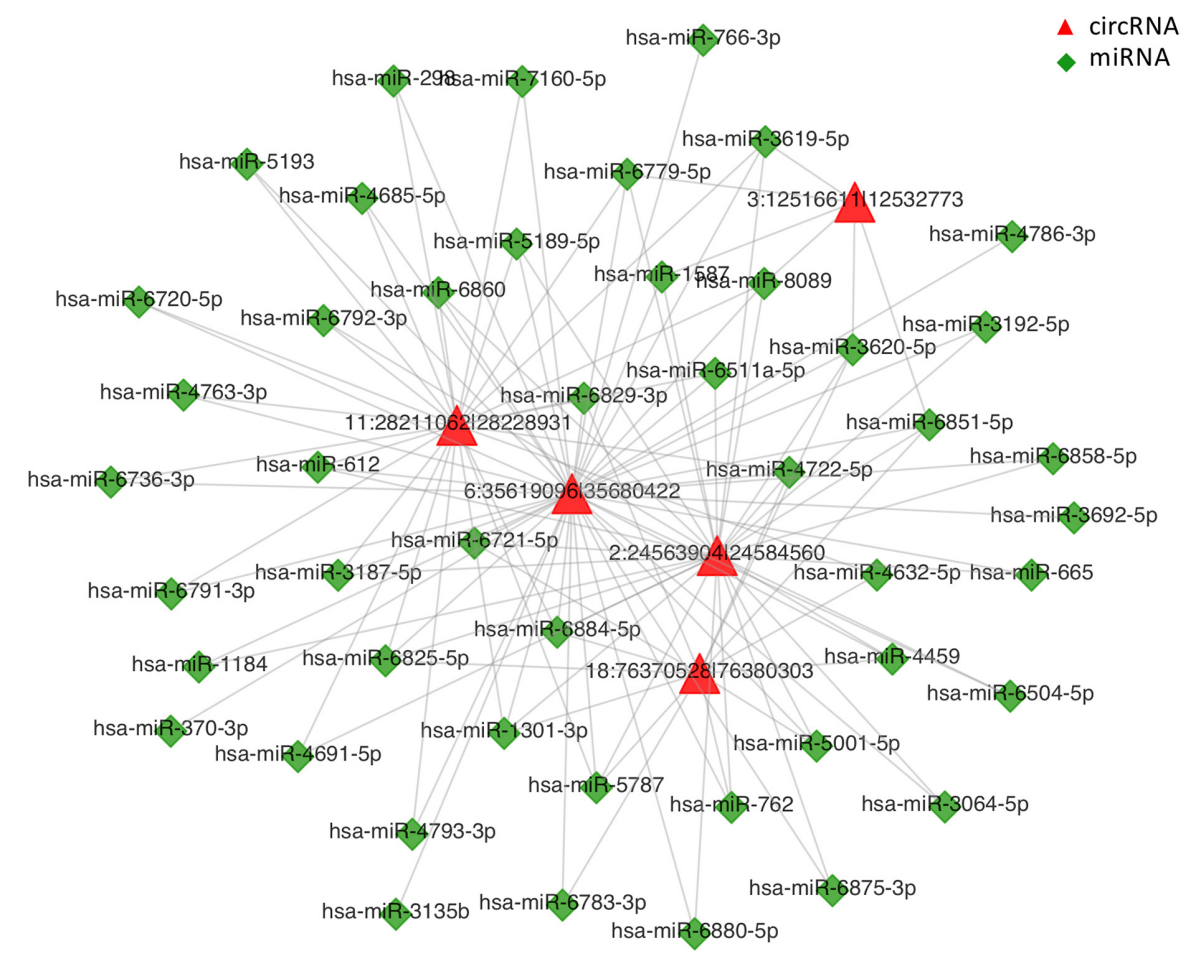
**CircRNA-miRNA network analysis.** The top 5 differentially expressed circRNAs and 47 predicted miRNAs were selected to generate a network map. The circRNA-miRNA network was constructed using bioinformatics online programs (starBase, circBase, TargetScan, and miRBase). The red triangle indicates circRNA and the green diamond indicates miRNA.

To further clarify the potential roles of circRNAs in the regulation of transcriptional and post-transcriptional levels, we constructed a circRNA-miRNA-mRNA interaction network using bioinformatics prediction software. The top 5 of DE-circRNAs were selected to construct the circRNA-miRNA interaction network as presented in Fig. 5. The circRNA-miRNA-mRNA interaction analysis for all DE-circRNAs was shown in Supplementary Table S5.

### *circDDX10-miR-1301-3p/miR-4660-SIRT3 interaction axis may participate in ovarian aging*

**Figure 6:**
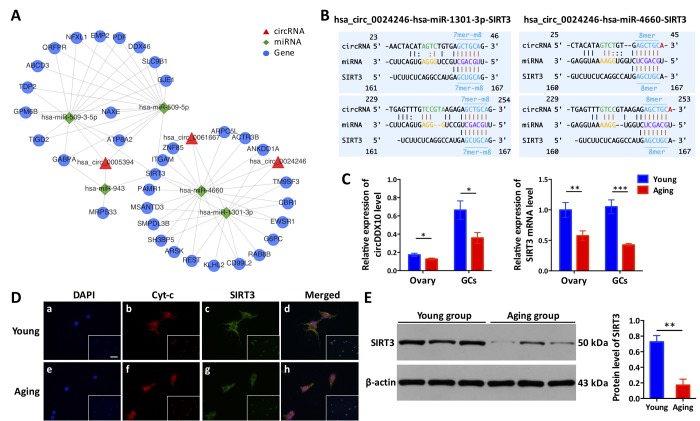
**The *circDDX10*-*miR-1301-3p/miR-4660*-*SIRT3* interaction axis is associated with ovarian aging.** (**A**) The predicted circRNA-miRNA-mRNA interaction network was predicted using bioinformatics online programs (starBase, circBase, TargetScan, and miRBase). The red triangle indicates circRNA, the green diamond indicates miRNA, and the blue circle indicates the targeted gene. (**B**) The predicted binding sites between *circDDX10*-*miR-1301-3p/miR-4660* and *miR-1301-3p/miR-4660*-*SIRT3* were exhibited. The matching types (in blue) and matching positions (in black and brown) were presented. The 7mer-m8, was an exact match to positions 2-8 of the mature miRNA (the seed + position 8), and the 8mer was an exact match to positions 2-8 of the mature miRNA (the seed + position 8) followed by an 'A'. (**C**) The expression of *circDDX10* and *SIRT3* in the ovarian cortex and granulosa cells (GCs) was examined by qPCR. (**D**) Co-localization of the SIRT3 protein to mitochondria in GCs. Patients (n = 5) per group and all samples were run in duplicate. Blue indicates 4′, 6-diamidino-2-phenylindole (DAPI) or nuclear staining. Red indicates cyto-chrome C (Cyt-c) and was used as a mitochondrial marker. Green indicates the SIRT3 protein. The scale bar is shown in the image of the bottom right corner (a) and equals 100 μm. (**E**) The expression of SIRT3 protein was determined using western blot. Proteins were isolated from GCs as described. Semi-quantitative analyses of protein levels were performed. Data indicate the mean ± SD, n = 3. *, *P* < 0.05; **, *P* < 0.01; ***, *P* < 0.001.

According to the results of the bioinformatics prediction, several targeted genes downstream (*SIRT3*, *PIK3CB*, and *CSE1L*) which may be associated with ovarian function (oocytes or GCs) were selected to construct the circRNA-miRNA-mRNA interaction networks (Fig. 6A and Supplementary Fig. S7). Here, we selected *circDDX10* as an example to further observe the influence of circRNAs on the expression of predicted targeted genes and proteins, to elucidate the *circDDX10*-*miR-1301-3p/miR-4660*-*SIRT3* interaction axis. The predicted binding sites between *circDDX10*-*miR-1301-3p*, *circDDX10*-*miR-4660*, *miR-1301-3p*-*SIRT3* and *miR-4660*-*SIRT3* were demonstrated in Fig. 6B. The relative expression of *circDDX10* and *SIRT3* mRNA in the ovarian cortex and GCs were examined by qPCR. Consistently, both the expression levels of *circDDX10* and *SIRT3* mRNA were significantly down-regulated with aging (n = 44, *P* < 0.05, Fig. 6C). Immunofluorescence showed that SIRT3 was co-localized with mitochondria (marked by Cyt-c) in human GCs. Additionally, the fluorescence intensity was higher in the young group compared with the aging group (n = 10, Fig. 6D). Furthermore, the average level of the SIRT3 protein was significantly higher in human GCs from the young group compared with the aging group (n = 6, *P* < 0.05, Fig. 6E).

## DISCUSSION

In this study, we first identified and annotated 48,220 human ovary-derived circRNAs using RNA-seq analysis. Most of these circRNAs were composed of protein-coding exons, which were similar to those in other human organs [26]. Several hundred circRNAs were significantly changed during aging. Of them, some were closely associated with biological processes involved in key signalling pathways, which may play essential roles in ovarian aging. Using a series of biological experiments, we further confirmed that ovary-derived circRNAs were resistant to RNase R digestion and were stably expressed in GCs. This indicates that circRNAs may be used as a potential biomarker for ovarian function and to predict the prognosis of reproductive outcomes in the future. Furthermore, construction of the circRNA-miRNA-mRNA interaction network provided a better understanding of the intrinsic relationship of different molecules involved in this complicated regulatory network.

Recently, it has been demonstrated that circRNAs play a role in both cellular senescence and cellular survival [27]. From a wide range of organisms, including *C. elegans* [13], *Drosophila* [12], mice [10], rats [28], monkeys [11], tree shrews [29], and pigs [30], a large number of circRNAs significantly changed during aging were identified. For example, the accumulation of circRNAs in the brain was discovered across different species compared to their linear isoforms [10,13,23,29]. This may account for the abundant enrichment of post-mitotic cells in neural tissues and the high stability of circRNAs in cells [13]. Consistent with these studies, we also identified many circRNAs differentially expressed during aging. We found that circRNAs identified in this study were distributed across the genome and most harboured 2-6 exons. The majority of circRNAs were exonic and approximately 700 nt in length, which were different from those in other organs or mammals [26,29]. These findings indicate that the distribution of circRNAs is tissue- and developmental stage-specific. Our study demonstrated that the majority (66.0%) of circRNAs identified are novel and uniquely expressed in the ovary and do not occur in other tissues. This may be attributed to low abundance in the ovary or biological differences between the two groups, which needs further investigation.

The structure of circRNAs are different from linear RNAs, as its 3' end is connected with the 5' end to form a covalently closed circular molecule. This unique structure without a polyA tail renders circRNA highly insensitive to ribonuclease. RNase R is an efficient 3' to 5' ribonucleic acid exonuclease, which can fully digest all linear RNAs, except for lariat RNAs and circRNAs, contributing the stability of circRNAs in cells and tissues [31,32]. The results of our study showed that circRNAs were more stable than their linear forms in cells, further confirming this distinct property. Given the advantage of stability, circRNAs are a promising biomarker for the diagnosis and prognosis of human diseases. For example, peripheral blood-derived circular RNA100783 could regulate phosphoprotein expression during CD28 related CD8(+) T cell aging, indicating its potential role as a novel biomarker for global immunosenescence [33]. Cheng et al. [34] revealed that the elevated expression of circRNA_103827 and circRNA_104816 derived from human GCs from follicular fluid were closely associated with a declining ovarian reserve and adverse reproductive outcomes. Moreover, circRNA_103827 has predictive performance for pregnancy outcomes after assisted reproductive technique cycles, indicating its potential as a new biomarker for IVF outcomes. However, the roles of circRNAs as potential biomarkers for ovarian aging needs further exploration.

Bioinformatics analysis is a method to better clarify the biological functions of circRNAs. Using GO and KEGG pathway annotation, we discovered several important biological processes and pathways, such as metabolic processes, regulated secretory pathways, oxidation-reduction processes, serine and threonine metabolism, steroid hormone biosynthesis, glycolysis/gluconeogenesis, and other important amino acid metabolism processes, which were highly associated with ovarian function during aging. Ovarian senescence is an age-dependent decline of female reproductive capacity, characterized by a reduction of the quality and quantity of oocytes and GCs [1,2]. Ovarian steroid production and subsequent local steroid-mediated signalling are critical for normal ovarian processes, including follicle growth, oocyte maturation, and ovulation [35]. By contrast, dysfunction of steroidogenesis, including oestrogens, androgens, and progestogens, could lead to ovarian dysfunction, such as polycystic ovarian syndrome, and even increase the risk of breast cancer [36,37]. In the present study, results of the KEGG pathway analysis showed that the circRNAs were highly associated with steroid hormone biosynthesis, indicating their potential effect on ovarian aging by regulating ovarian steroidogenesis, although the specific mechanisms remain to be elucidated.

Previous studies confirmed that most circRNAs, located in the cytoplasm, can act as miRNA sponges to regulate gene expression and protein translation [38–41]. According to our results, circRNAs harboured numerous well-conserved miRNA binding sites, indicating their potential functions by interacting with miRNAs. By constructing a circRNA-miRNA-mRNA regulatory network using bioinformatics, we uncovered the underlying mechanism by which circRNAs are involved in the process of ovarian aging. For example, as shown in Fig. 6A, we determined that the *circDDX10*-*miR-1301-3p/miR-4660*-*SIRT3* interaction axis is involved in the regulatory network. Recently, SIRT3, as one of the sirtuin family members, is associated with aging. Several studies confirmed the crucial roles of SIRT3 in ovarian aging [42–44]. Our preliminary study revealed that the expression of SIRT3 was significantly down-regulated during aging, as demonstrated by immunofluorescence and western blot analyses (Fig. 6C, D), which was consistent with previous studies. Furthermore, we showed that the level of *circDDX10* was also down-regulated during aging, consistent with the expression pattern of SIRT3. Given the potential binding sites between *circDDX10*-*miR-1301-3p/miR-4660* and *miR-1301-3p/miR-4660*-*SIRT3*, we propose that circDDX10 may promote the expression of *SIRT3* through the absorption of *miR-1301-3p/miR-4660*. However, the underlying mechanisms need further investigation.

In recent years, several studies identified the expression profiles of circRNAs in the ovaries of *Drosophila* [23], goats [24] and honey bees [25]. However, to the best of our knowledge, this study reported, for the first time, the circRNA profiles of human ovarian tissues and identified hundreds of DE-circRNAs during ovarian aging. The unique biological properties of circRNAs were further confirmed, indicating their potential roles as biomarkers of ovarian aging. The construction of a circRNA-miRNA-mRNA interaction network will provide a better understanding of the complex regulatory relationship and mechanism of ovarian aging.

There are several drawbacks inherent to our study. First, the major limitation of our study is the relatively small sample size included for RNA-seq and validation. Second, due to ethical issues, it is not possible for us to collect complete normal ovarian tissues from a healthy population. Therefore, the influence from the gynecological diseases themselves could not be neglected. Moreover, to some extent, the existence of biological differences could introduce bias to our data. Finally, we did not perform further experiments to confirm the regulatory relationship among circRNAs, miRNAs and mRNAs and their roles in ovarian aging.

## CONCLUSIONS

In conclusion, we first performed the genome-wide identification of human ovary circRNAs by RNA-seq, and found that they are abundant and spatiotemporal-specific during ovarian aging. Most of the exonic circRNAs harbour miRNA binding sites and some may play key roles in ovarian function by sequestering miRNAs, as well as other mechanism. The *circDDX10*-*miR-1301-3p/miR-4660*-*SIRT3* interaction axis may be involved in the regulatory network of ovarian aging. However, more experiments are needed to confirm this relationship. The diversified biological functions of circRNA in the ovary need further exploration. More studies on the mechanisms of circRNA, particularly during important biological events, such as the maturation and meiosis of the oocyte, will help identify a method to slow the process of ovarian senescence.

## MATERIALS AND METHODS

### Sample collection and preparation

Patients undergoing benign gynecological operations at the Department of Obstetrics and Gynecology, Tongji Hospital Affiliated to Tongji Medical College from February 2017 and October 2017 were recruited in this study. The mean age was 35.4 ± 9.3 years old, ranging from 20 to 49 (n = 44). Patients with acute infectious diseases, malignant tumours, hereditary diseases and systemic immune diseases were excluded. Moreover, patients with polycystic ovary syndrome, premature ovarian failure, endometriosis and other reproductive endocrine diseases, such as thyroid disease, diabetes, adrenal disease, *etc.*, were further excluded. The basic information of the patients included for RNA-seq (n = 6) was presented in Table 2. A small piece of the ovarian cortex on the normal side of the patient was excised during the operation and then immediately washed with aseptic saline 2 times to rinse off the surface blood and residual tissues. Part of the ovarian sample was fixed with 4% paraformaldehyde for histological analysis. The rest of the specimen was then cut into a 0.5 × 0.5 × 0.1 cm slices and preserved in a 2 ml cryopreservation tube and then quickly put into a liquid nitrogen tank within 2 min. This study was approved by the ethics committee of Tongji Medical College, Huazhong University of Science and Technology (NO. 2016 (04)), Wuhan, China. Informed consent was obtained from all participants in this study.

**Table 2:** Basic information of the included participants in this study.

**No.**	**Age**	**BMI (kg/m^2^)**	**Serum reproductive hormones evaluation**			
			**AMH (ng/ml)**	**FSH (IU/l)**	**LH (IU/l)**	**E_2_ (pg/ml)**
1	25	22.31	4.76	5.57	4.77	45
2	26	21.74	6.69	5.59	5.73	61
3	28	20.70	7.44	3.76	5.31	43
4	44	22.76	1.52	11.6	5.66	60
5	45	26.17	0.57	9.79	6.66	37
6	46	26.78	0.52	6.77	5.74	46

AMH, Anti-Müllerian hormone; BMI, body mass index; E_2_, estradiol; FSH, follicle-stimulating hormone; LH, luteinizing hormone; P, progesterone; PRL, prolactin; T, testosterone.

### CircRNA high-throughput sequencing and biological information annotation

### *Total RNA isolation and rRNA-depletion*

Histological analysis using haematoxylin-eosin (HE) staining was performed on the ovarian tissues to confirm the suitability of the samples. Only those with normal morphological structures in accordance with reproductive ages were eligible (As shown in Supplementary Fig. S8). The histomorphological assessment was confirmed by a sophisticated pathologist in the laboratory. The total RNA from six ovarian cortex samples (young and aging group, each with 3 samples) was extracted using TRIzol reagent (Life Technologies, CA, USA) and then treated with DNase (Takara, Dalian, China) following the manufacturer’s instructions. RNA integrity was assessed using an Agilent Bioanalyzer 2100 (Agilent Technologies, Santa Clara, CA, USA). The quantity (ng/ml) and purity (260/280 and 260/230 ratios) of total RNAs were assessed with a NanoDrop 2000 spectrophotometer (Thermo, Waltham, MA, USA) and denaturing agarose gel electrophoresis. Total RNAs were incubated with RNase R (Ribonuclease R, *E. coli*, Cat. No. RNR07250, Epicentre, Madison, Wisconsin, USA) 1 IU enzyme/1 μg RNA, at 37°C for 10 min for rRNA-depletion, followed by heat inactivation at 95°C for 3 min. The RNAs were then preserved at -80°C until the next step.

### *CircRNA library construction and Illumina deep sequencing*

CircRNA-Seq libraries were generated using the Tru Seq RNA LT Sample Prep Kit v2 (Illumina, San Diego, CA, USA) following the manufacturer’s instructions. The libraries were sequenced using an Illumina Hiseq 3000 platform in PE150 sequencing mode with a Paired-End module (at a depth of 50 million reads) by Genergy Biotechnology Co., Ltd. (Shanghai, China).

### *Sequence mapping and circRNA annotation*

Sequence reads were first mapped using TopHat against the GRCh37/hg19 human reference genome with the UCSC Genes annotation. For all raw sequencing data for each sample, adapter reads and low-quality reads were removed using Trim Galore. CIRCexplorer2 software was applied to obtain back-spliced junction reads for circRNA prediction. The expression of circRNAs was quantified using the number of reads spanning back-spliced junctions (circular reads). The relative expression of circRNAs was denoted as BSRP (back-spliced reads per million mapped reads), using circular reads normalized to per million mapped reads.

### *Differential expression analysis*

DE-circRNAs in the young and aging groups were identified using the DESeq2 software with a *t* test *P* value < 0.05 and fold change > 2. The top 200 DE-circRNAs were log_2_ transformed, gene mean centred and visualized as a heatmap using the Multi Experiment Viewer (www.tm4.org). Volcano plots were generated using gglpot2 in R (https://cran.r- project.org/web/packages/ggplot2/index.html).

### *Functional annotation, target miRNA and interaction network prediction*

For functional annotation, all parental genes of the DE-circRNAs were subjected to GO (www.geneontology.org) and KEGG (www.genome.jp/kegg) pathway enrichment analyses using DAVID Bioinformatics Resources 6.8 (david.ncifcrf.gov/home.jsp). The *P* value was calculated using a hypergeometric test and corrected by Benjamini-Hochberg adjustment. We regarded the negative logarithm (base 10) as the enrichment score that indicated the significance of correlation. The prediction of potential circRNA-miRNA binding sites was performed using miRanda (www.microrna.org/microrna/home.do). The circRNA-miRNA-mRNA interaction analysis was performed for all DE-circRNAs. The enrichment score was the proportion of combined miRNAs in all circRNA-binding miRNAs/the proportion of mRNA-binding miRNAs in all referenced miRNAs and was measured according to the common combined miRNA between the mRNA 3’UTR sequences and cirRNA sequences. The cirRNA-miRNA-mRNA interaction axis was considered significant using a hypergeometric test (FDR value < 0.01, enrichment score ≥ 20), and the predicted target genes of the DE-circRNAs were further subjected to GO term and KEGG analyses. The gene network analysis was performed using Cytoscape (version 3.6.0, United States).

### cDNA synthesis, PCR, electrophoresis and quantitative real-time PCR (qRT-PCR)

Total RNA was reverse transcribed using random primers with the PrimeScript RT reagent kit (RR047A, Takara, Dalian, China) according to the manufacturer’s protocol. We randomly selected 22 DE circRNAs (Fold change > 2, *P* < 0.05), including eight up-regulated, eight down-regulated circRNAs and six without significant differences, for subsequent validation. We designed at least three paired divergent primers encompassing circRNA-specific back-splice junctions for each of the candidate circRNA. Details of the primer sequences are summarized in Supplementary Table S6. Only primers achieving a single peak in the melting curve were considered proper for qRT-PCR validation. The qRT-PCR was performed using SYBR Premix Ex Taq II (Takara, Dalian, China) on a Roche LightCycler96 thermocycler according to the manufacturer’s instructions (95°C for 3 min and 40 cycles of 95 °C for 10 s, annealing temperature (58-62 °C) for 30 s, and 72 °C for 30 s, followed by 72 °C for 1 min). The relative expression of candidate circRNAs was normalized to glyceraldehyde-3-phosphate dehydrogenase (*GAPDH*) and then calculated using the 2^-ΔΔCt^ method [45]. All experiments were repeated in triplicate and presented as the means ± SEM. Finally, PCR products were validated by 2% agarose gel electrophoresis. To confirm the junction sequence of circRNAs, PCR products were gel purified using the TIANgel Midi Purification Kit (Beijing, China) according to manufacturer’s instructions and submitted for Sanger sequencing at Icongene Co. Ltd (Wuhan, China).

### Genomic DNA extraction

Genomic DNA (gDNA) of the ovary samples was extracted using the TIANamp Genomic DNA Kit (DP304, TIANGEN BIOTECH, Beijing, China) in accordance with the manufacturer’s instructions. The gDNA fraction was immediately transferred to a 2 ml microfuge tube and stored at –80 °C until further processing.

### RNase R treatment

Total RNA was incubated with RNase R (1 IU enzyme per μg RNA) in 1× RNase R buffer at 37 °C for 15 min, and then quickly placed on ice. Control group was treated with equal volume of ddH_2_O. DNase digestion, cDNA synthesis and qRT-PCR procedures were performed as described above. qRT-PCR Ct values were calculated automatically and ∆Ct was termed as Ct (RNase R treatment) − Ct (mock treatment). The expression of circRNAs and mRNAs before the RNase R treatment were normalized as 1. 2^−∆Ct^ was used to compare the expression of circRNAs and mRNAs after RNase R digestion.

### PolyA^+^ RNA extraction

PolyA^+^ RNA was enriched using the kit (Magnosphere^TM^ UltraPure mRNA Purification Kit, Takara, Dalin, China), according to the manufacturer’s protocol manual. The polyA^+^ RNA fraction was immediately transferred to a 2 ml microfuge tube and stored at –80 °C until further processed.

### Isolation, purification and identification of GCs

On the oocyte retrieval day, all follicular fluid samples from the same patient were pooled after cumulus–oocyte complexes were isolated for conventional in vitro fertilization (IVF) or intracytoplasmic sperm injection (ICSI) procedures. GCs were individually isolated from follicular fluid samples using similar methods as previously described [43]. Briefly, the follicular fluid sample of each person was centrifuged and resuspended in 1× phosphate-buffered saline (PBS). Then, it was slowly placed on a 50% Percoll gradient (GE Healthcare Life Sciences, Piscataway, NJ, USA) and centrifuged at 400× g for 30 min at 4 °C. The cells in the middle layer were carefully collected, washed and resuspended in PBS. To confirm the purity of GCs, cells were stained with antibodies against FSHR (follicle stimulating hormone receptor; 1: 100; ab113421, Abcam, Cambridge, MA, USA) according to a previous study [46]. The harvested GCs were stored in TRIzol reagent (Life Technologies, CA, USA) at -80 °°C until RNA extraction.

### Immunofluorescence

The expression of SIRT3 in the cultured GCs was investigated by immunofluorescence according to a previous study [43]. The cells in the 6-well plates were fixed to poly-lysine slides with 4% paraformaldehyde for 30 min at room temperature and then washed three times with Dulbecco’s PBS. The slides were incubated in blocking buffer (10% normal goat serum) for 10 min at 37 °C. The localization of SIRT3 to the mitochondria was detected using co-immunofluorescence with cytochrome C as a marker of mitochondria. Then, the slides were incubated overnight in a humidified chamber at 4°C with anti-SIRT3 rabbit polyclonal antibody (1:100; ab45067, Abcam, Cambridge, MA, USA) and a cytochrome C primary antibody (1:500; ab110325, Abcam, Cambridge, MA, USA) overnight at 4°C. After washing with PBS three times, the slides were incubated with the appropriate secondary antibodies (1:400) for 2 h at 37 °C. The slides were further washed three times with PBS and were counterstained with DAPI (sc-3598, Santa Cruz Biotech, CA, USA) to visualize nuclei. Images were obtained using a Leica DMI3000B microscope. A negative control using a SIRT3 blocking peptide (Abgent, BioCore, Alexandria, New South Wales, Australia) was also prepared and imaged. The expression of SIRT3 in the cultured cumulus cells was served as a positive control.

### Western blotting

The cultured GCs were lysed using RIPA protein extraction reagent (Beyotime, Beijing, China) supplemented with a protease inhibitor cocktail (Roche, CA, USA). Protein quantification was determined by the Bradford method (Beyotime, Shanghai, China). Approximately 40 μg of protein extract was separated by 10% SDS-polyacrylamide gel electrophoresis (SDS-PAGE) and then transferred to a nitrocellulose membrane (Sigma-Aldrich, MO, USA). After blocking for 2 h, membranes were incubated with SIRT3 antibody (1:1000; as used for immunofluorescence) at 4 °C overnight. Membranes were washed and incubated for 1.5 h with HRP conjugated secondary antibodies (1:2000; ab6721, Abcam, Cambridge, MA, USA). ECL chromogenic substrate was used to visualize the bands, and the band intensity was measured using Image J (version 1.49s, National Institutes of Health, USA). β-Actin was used as a control, and a negative control without primary antibody was also prepared.

### Statistical analysis

Statistical analyses were performed using SPSS Statistics (version 23.0; IBM, Armonk, NY, USA) and GraphPad Prism 6.0 (version 6.0c; GraphPad Software, Inc., San Diego, CA, USA). All the data are displayed as the mean ± SEM for triplicate independent measurements. Student's *t* test was used to assess the differences between experimental groups. Differences with *P* values < 0.05 were considered statistically significant.

## Supplementary Material

Supplementary Figures

Supplementary Table S1

Supplementary Table S2

Supplementary Table S3

Supplementary Table S4

Supplementary Table S5

Supplementary Tables 6 and 7
