# Supplementary material for: Identification and characterization of human ovary-derived circular RNAs and their potential roles in ovarian aging
Source: Aging (Albany NY). 2018 Sep 23;10(9):2511–34. doi: 10.18632/aging.101565 (PMC6188495; doi:10.18632/aging.101565)
Supplement: Supplementary Figures [file aging-10-101565-s001.pdf]

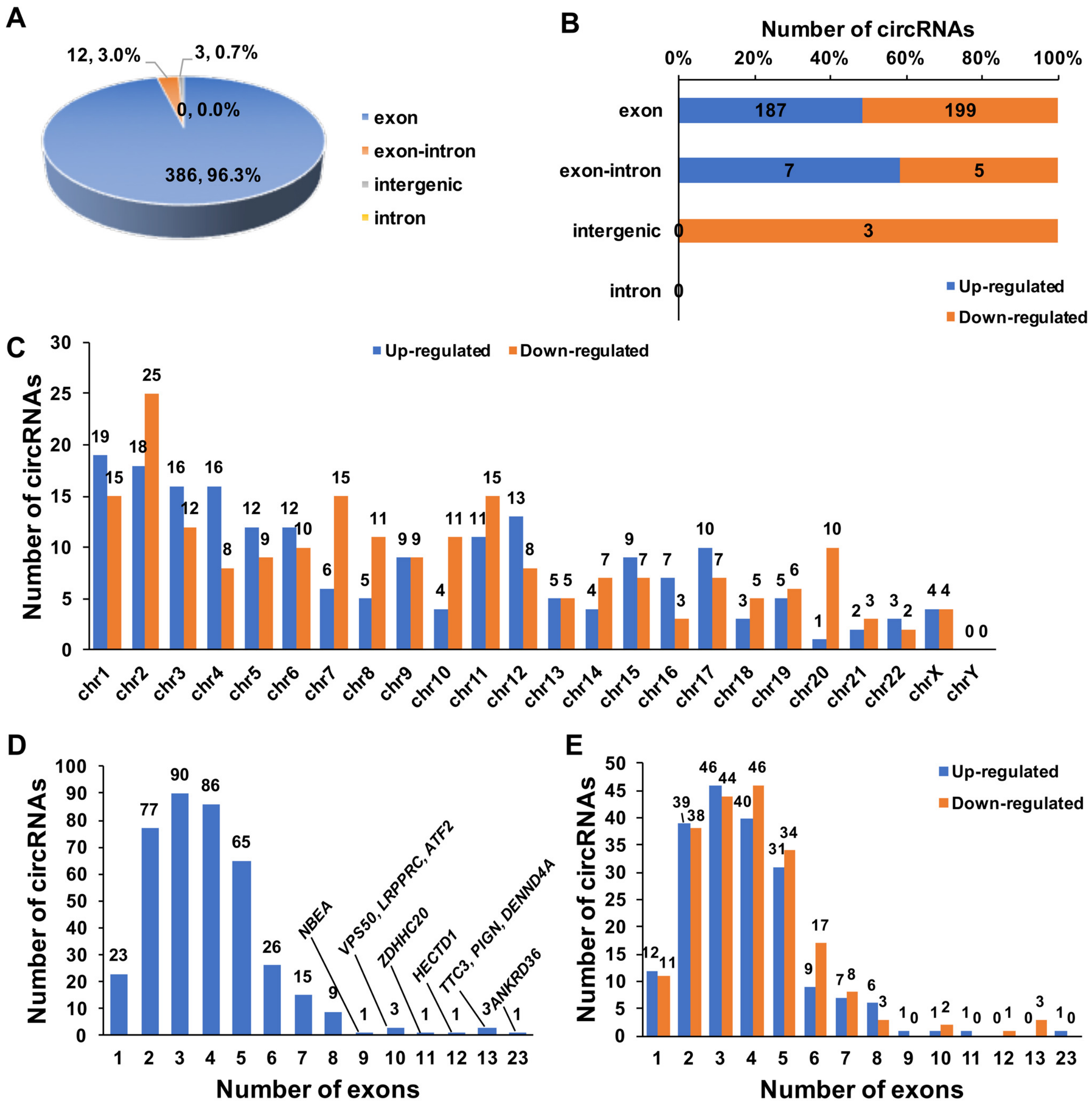

Figure S1. Overview of the DE-circRNAs in human ovarian tissues. (**A**) The proportion of different types of circRNAs among the DE-circRNAs. (**B**) The proportion of DE-circRNAs among different types of circRNAs. (**C**) The distribution of DE-circRNAs in human genome. (**D**, **E**) The distribution of exons among the DE-circRNAs.

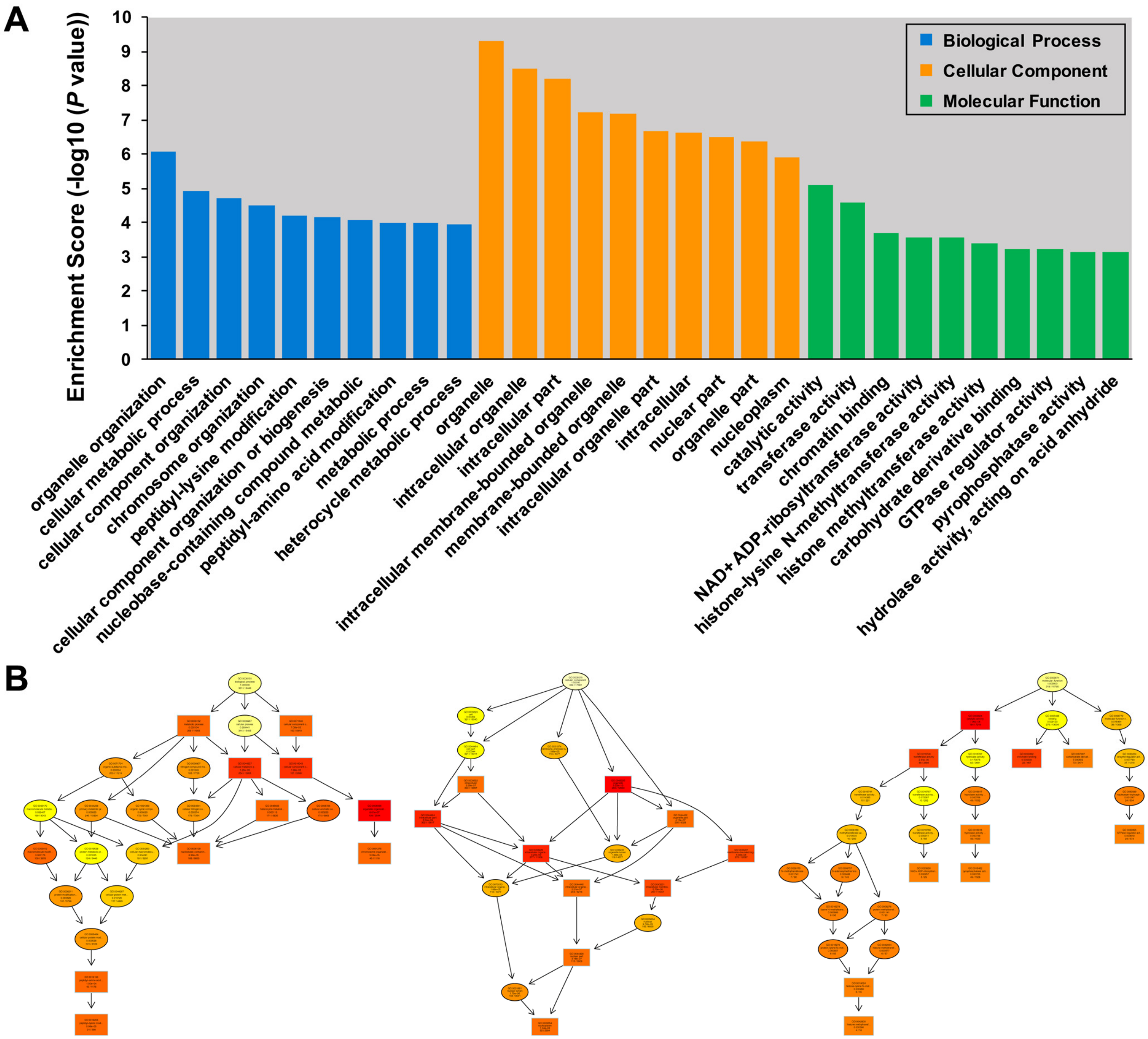

Figure S2. Gene Ontology (GO) annotation of the targeted genes. Histogram (**A**) and dendrogram (**B**) of the Top 10 of the GO terms regarding each category (Biological process; cellular component and molecular function).

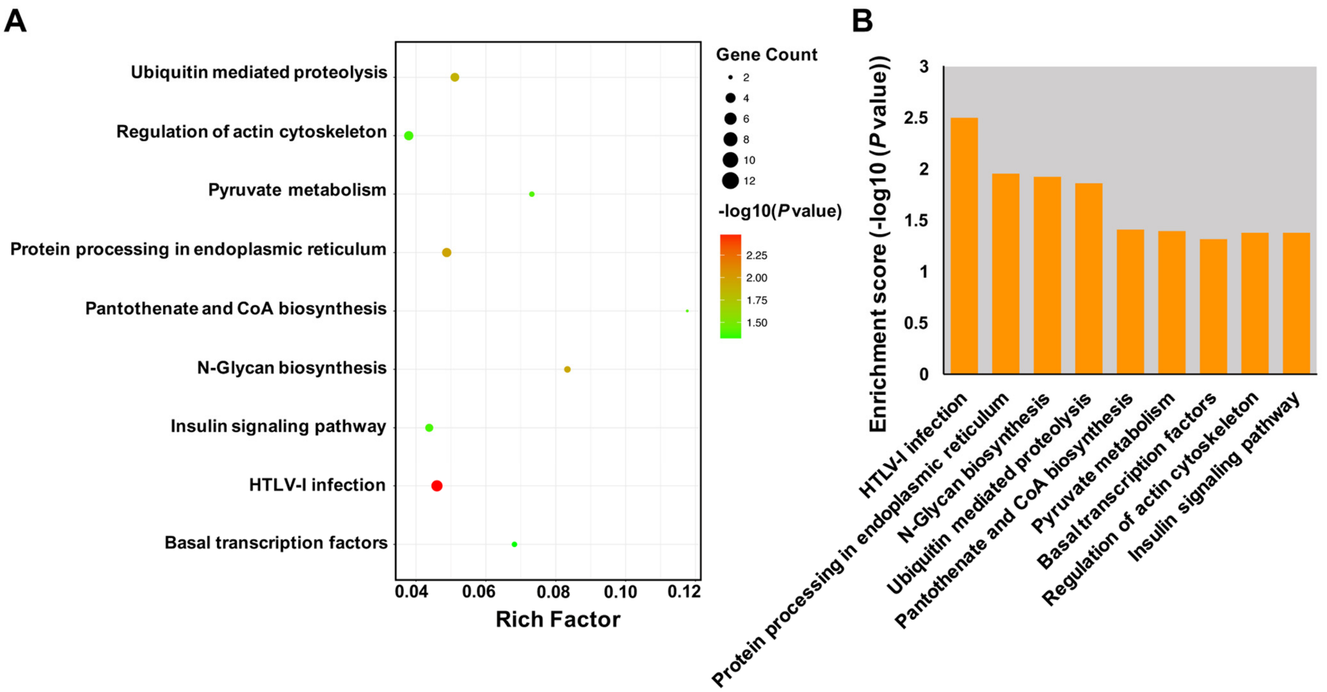

Figure S3. Kyoto Encyclopedia of Genes and Genomes (KEGG) pathway annotation of the targeted genes. Scatter diagram (**A**) and histogram (**B**) of the Top 9 of the KEGG pathways. Circle indicates the gene count; The darker (red) color indicates that the enrichment score is higher while the lighter (green) color indicates that the enrichment score is lower. The enrichment score was calculated as –log10 (*P* value).

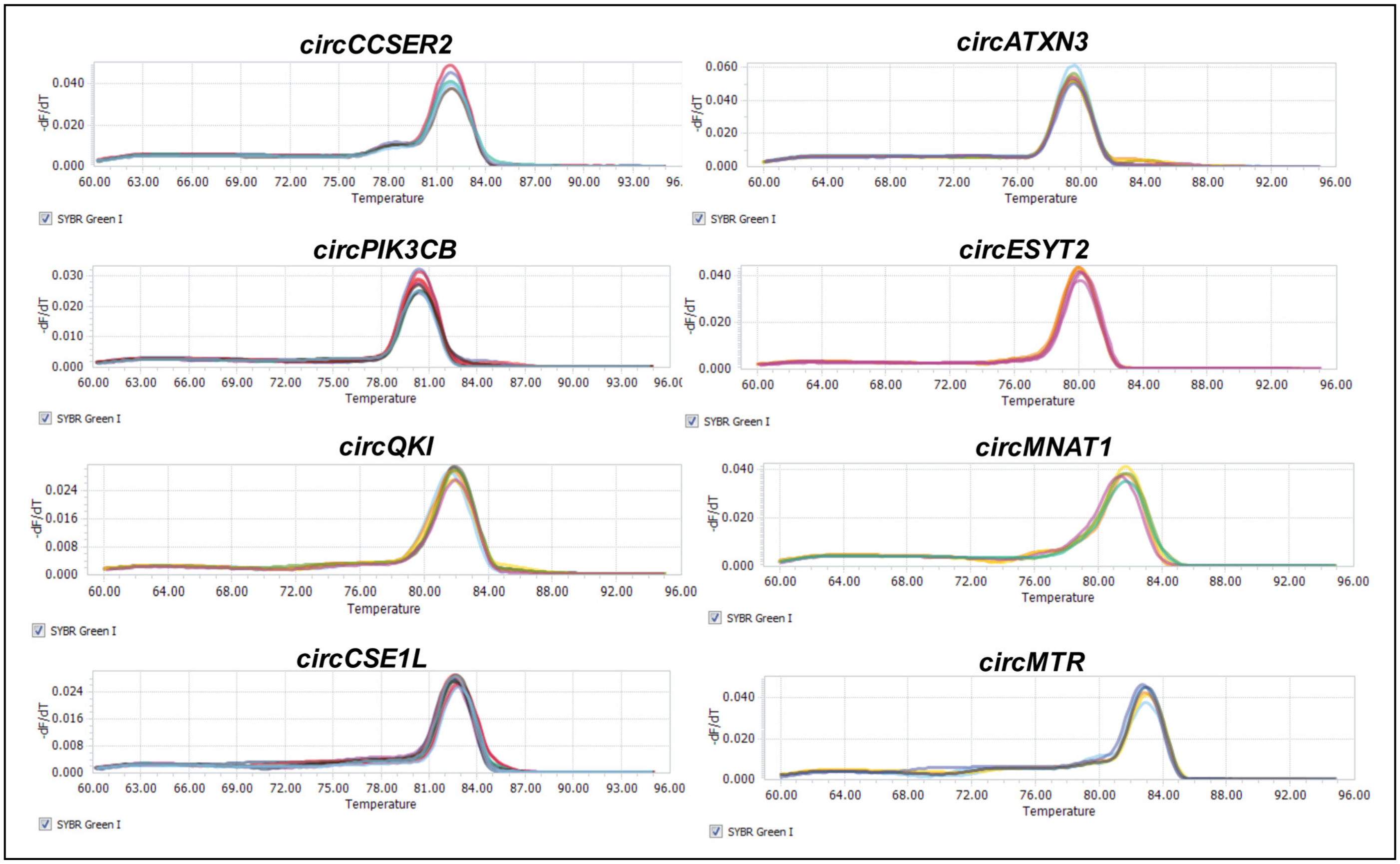

Figure S4. Melting curves of the randomly selected circRNAs for qPCR validation. The experiments were performed by a Roche LightCycler® 96. All of the melting curves were demonstrated as single peaks.

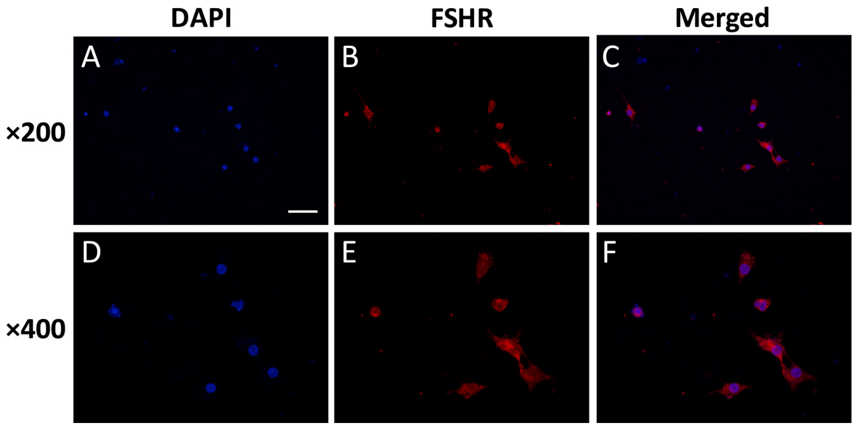

Figure S5. Purity identification of the harvested granulosa cells (GCs). Fluorescence microscopy images of GCs isolated from human follicular fluid. Images of GCs stained with DAPI, FSHR (marker of GCs) and their merged image are shown. The scale bar represents 20 µm. APC indicates allophycocyanin; DAPI, 4′, 6-diamidino-2-phenylindole; FSHR, Follicle Stimulating Hormone Receptor. Original magnification ×200 and ×400. Scale bar is representatively shown in (**A**), and equals 50 µm (**A**, **B** and **C**) and 25 µm (**D**, **E** and **F**).

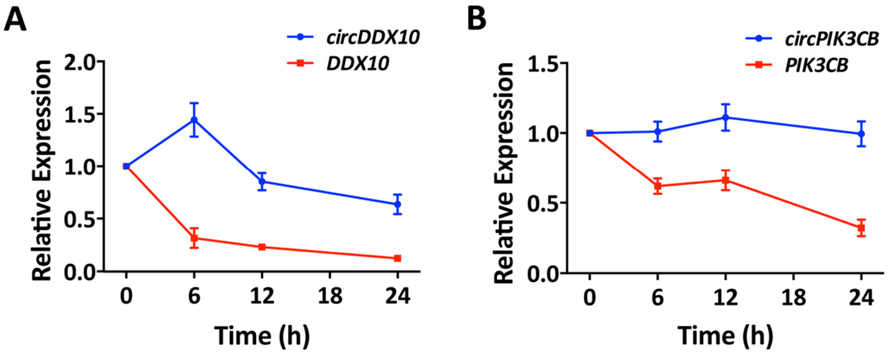

Figure S6. Ovary-derived circRNAs can be stably expressed in granulosa cells (GCs). The relative expression of circRNAs and their linear counterparts were determined by qRT-PCR and measured to GAPDH. Ovary- derived circRNAs can be stably detected in human GCs from follicular fluid in 24 h, while their linear forms degraded rapidly. All data were performed triplicated and indicated as mean ± SD.

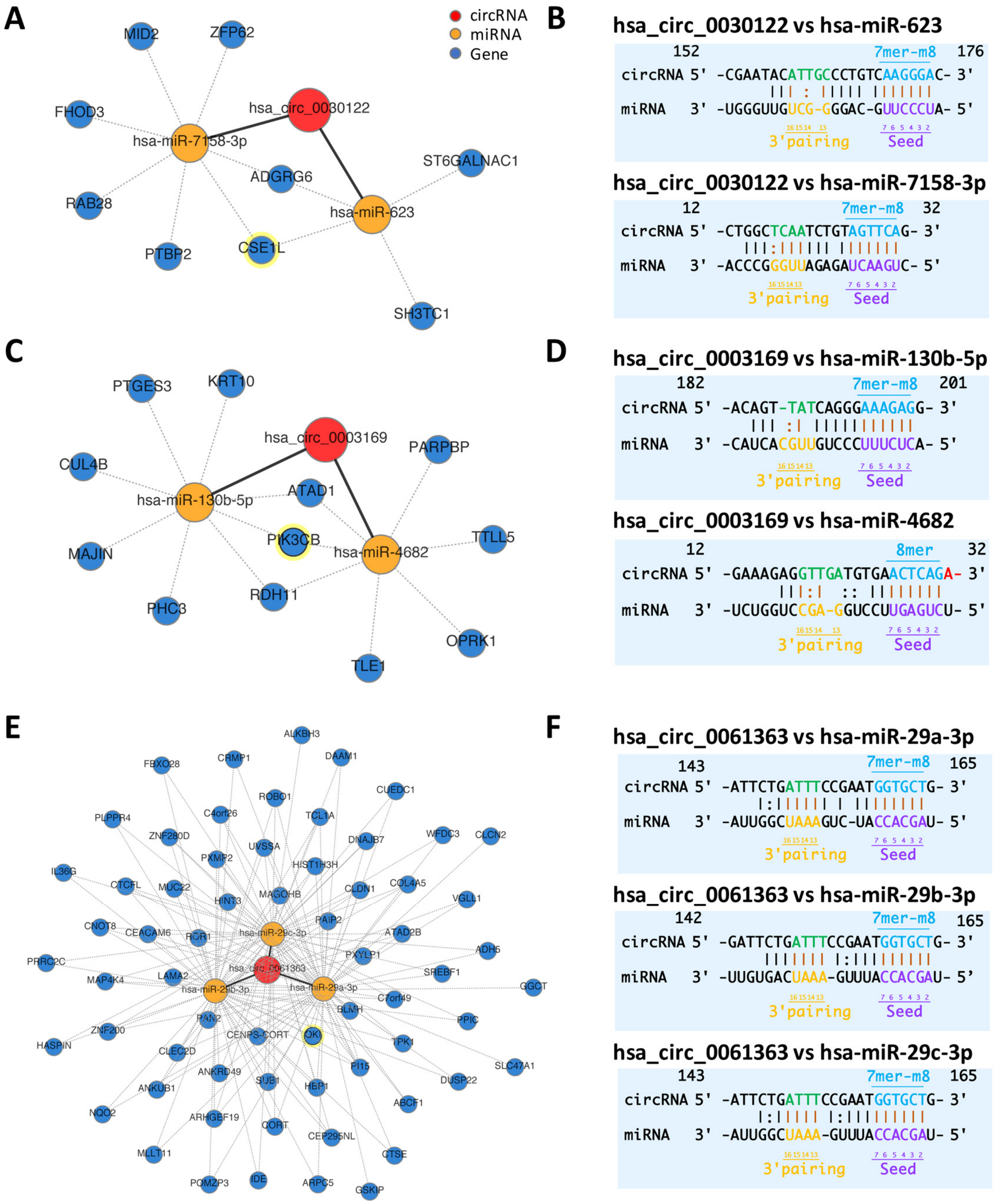

Figure S7. circRNA-miRNA-mRNA interaction network analysis and predicted binding sites. (**A**, **B**) *circFAM185BP-hsa-miR-130b-5p/hsa-miR-4682-mRNAs* and predicted binding sites. (**C**, **D**) *circVWA8-hsa-miR-623/hsa-miR-7158-3-mRNAs* and predicted binding sites. (**E**, **F**) *circLTN1-hsa-miR-29a-3p/hsa-miR-29b-3p/hsa-miR-29c-3p-mRNAs* and predicted binding sites. The matching types (in blue) and matching positions (in black and brown) were presented. The predicted circRNA-miRNA-mRNA interaction network were predicted through bioinformatics online programs (starBase, circBase, TargetScan, miRBase). 7mer-m8: an exact match to positions 2-8 of the mature miRNA (the seed + position 8); 8mer: an exact match to positions 2-8 of the mature miRNA (the seed + position 8) followed by an 'A'.

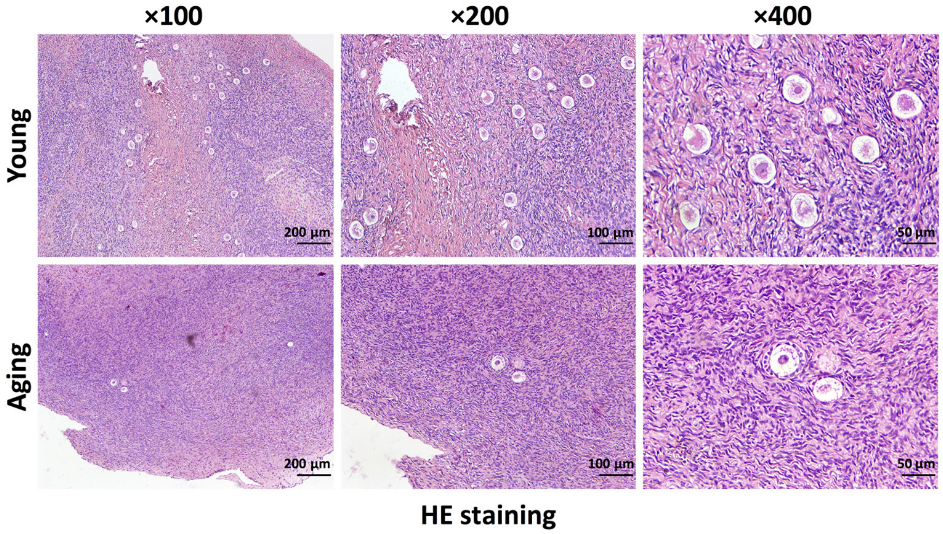

Figure S8. Histological analysis of the ovarian tissues. Hematoxylin-eosin (HE) staining was performed on the ovarian tissues of young and aging groups. From left to right, image magnifications of ×100, ×200, and ×400 are displayed. Scale bar = 200 µm, 100 µm and 50 µm, respectively.
