# Supplementary material for: Identification and characterization of human ovary-derived circular RNAs and their potential roles in ovarian aging
Source: Aging (Albany NY). 2018 Sep 23;10(9):2511–34. doi: 10.18632/aging.101565 (PMC6188495; doi:10.18632/aging.101565)
Supplement: Supplementary Tables 6 and 7 [file aging-10-101565-s007.pdf]

## SUPPLEMENTARY TABLES

**Table S1.** Detail information of all the circRNAs identified in this study – see Full Text version
**Table S2.** Detail information of the differentially expressed circRNAs identified in this study – see Full Text version
**Table S3.** Details of the GO enrichment with statistical significance – see Full Text version
**Table S4.** Details of the KEGG enrichment with statistical significance – see Full Text version
**Table S5.** The circRNA-miRNA-mRNA interaction analysis for all DE-circRNAs – see Full Text version

**Table S6. Primer sequences used in this study.**

| Transcript ID or Position | Gene Symbol | Exon Composition | Primer Sequence | AT (℃) | Product Length (bp) |
|---|---|---|---|---|---|
| **Up-regulated circRNAs** | | | | | |
| hsa_circRNA_0018996 | *CCSER2* | 4-9 | F: 5'-TGTCATTGCCATCCAGTC-3'<br>R: 5'-AGCGGTTCATATTGTCACA-3' | 61 | 342 |
| hsa_circRNA_0008016 | *FGFR1* | 3 | F: 5'-CCTACTTCTCCGTCAATGTT-3'<br>R: 5'-CAGCCAGTTGATGCTCTG-3' | 60 | 134 |
| hsa_circRNA_0003169 | *FAM185BP* | 4-6 | F: 5'-GGTTGATGTGAACTCAGAAG-3'<br>R: 5'-GAAGACGAATCTACTGTGATG-3' | 57 | 126 |
| hsa_circRNA_0005328 | *QKI* | 2-3 | F: 5'-CTTGGACCTAGAGGACTTAC-3'<br>R: 5'-CAGCATCAGGCAATTCTG-3' | 60 | 180 |
| hsa_circRNA_0008421 | *FBN1* | 3-6 | F: 5'-CCAGTGTGAAAGAGACCCAATG-3'<br>R: 5'-CCGGCAAATGGGGACAATAC-3' | 60 | 108 |
| hsa_circRNA_0017105 | *NID1* | 5-9 | F: 5'- GGCGAACCTGCTATGTTTTCA-3'<br>R: 5'-GCGCCCGTGGTTCATTACTA-3' | 55 | 284 |
| hsa_circRNA_0004431 | *PIK3CB* | 15-17 | F: 5'-ATGAAGCCTTTGTGGCTGGT-3'<br>R: 5'-GAGTGCTTCAACCGAAGATCC-3' | 57 | 170 |
| hsa_circRNA_0029434 | *ADGRD1* | 9-13 | F: 5'-AGCACAGATTGACCTTCTT-3'<br>R: 5'-TCGATGAGACTCAGTACCA-3' | 52 | 100 |
| **Down-regulated circRNAs** | | | | | |
| chr3:12516611-12532773 | *TSEN2* | 7-9 | F: 5'-GAGAGGAGTGACCAAGACGAT-3'<br>R: 5'-AACATCCCAGAGCATAGACCAA-3' | 55 | 110 |
| chr14:92088730-92096838 | *ATXN3* | 2-4 | F: 5'-CAGTCCAGAGTATCAGAGG-3'<br>R: 5'-CTTCTTGCTTCCTGTTGTAA-3' | 55 | 198 |
| hsa_circRNA_0017160 | *MTR* | 16-18 | F: 5'-AAGCCATTCGAGAAGCAAT-3'<br>R: 5'-GTGTTCCTCCATTCCAGTC-3' | 61 | 326 |
| hsa_circRNA_0004244 | *MNAT1* | 4-7 | F: 5'-ACTGGCACCTATTCACAAG-3'<br>R: 5'-TCCTGTCGTTCCACTTCT-3' | 61 | 262 |
| hsa_circRNA_0020611 | *SIRT3* | 2-4 | F: 5'-TCTACACGCAGAACATCG-3'<br>R: 5'-CACTTCCAACAACACTTGAA-3' | 59 | 196 |

| | | | | | |
|---|---|---|---|---|---|
| hsa_circRNA_0083220 | *ESYT2* | 2-7 | F: 5'-TAACTGGACAGGACTGACGAAT-3'<br>R: 5'-CCGCACGGCTGGTTCTATA-3' | 61 | 165 |
| hsa_circRNA_0060762 | *CSE1L* | 19-23 | F: 5'-AGAGCATGATCCTGTAGGT-3'<br>R: 5'-GGCATGTGCTCTATTATACTG-3' | 60 | 186 |
| hsa_circRNA_0000983 | *NCOA1* | Intron 2-3 + Exon 3-4* | F: 5'-AACAGGAGTTCTTCTGCTAG-3'<br>R: 5'-CACACCACTGTTTGTAAGC-3' | 60 | 333 |
| hsa_circRNA_0024246 | *DDX10* | 7-10 | F: 5'-GGAAATCAACACCCCTGCCA-3'<br>R: 5'-CGACCATGGAGTGCAAGGAT-3' | 60 | 204 |
| hsa_circRNA_0061363 | *LTN1* | 2-3 | F: 5'-GGTGCTGCGGAAACTTTCAA-3'<br>R: 5'-GTCACTCTGAGATGTTCCA-3' | 63 | 235 |
| **Common-expressed circRNAs** | | | | | |
| hsa_circRNA_0058213 | *ARPC2* | 6-8 | F: 5' CAGTAGTCTTCAGCACAGT-3'<br>R: 5'-ACATTGTATCAGAGGAGGTT-3' | 52 | 146 |
| hsa_circRNA_0006166 | *RGPD2* | 13-14 | F: 5'-TCAGGCATCAGAAATTGTTG-3'<br>R: 5'-GGATCAATAGGTTCAGGAATAC-3' | 55 | 267 |
| hsa_circRNA_0000442 | *MED13L* | Exon 2 + Intron 2-3* | F: 5'-TTGCAGCAGCATTGTAGA-3'<br>R: 5'-CCACCAGAATATCCATAACTC-3' | 60 | 250 |
| hsa_circRNA_0005078 | *BMPR2* | 12 | F: 5'-ATCAAAGCCCAGAAGAGCAC-3'<br>R: 5'-AGGACCAATTTTTGGCACAC-3' | 60 | 120 |
| hsa_circRNA_0001181 | *BACH1* | 2-4 | F: 5'-CGCTGTCGCAAGAGAAAAC-3'<br>R: 5'-AGGCAAAAACCGAGTTCTCA-3' | 60 | 151 |
| hsa_circRNA_0001821 | *PVT1* | 2 | F: 5'-GACTCTTCCTGGTGAAGCATCTGAT-3'<br>R: 5'-TACTTGAACGAAGCTCCATGCAGC-3' | 60 | 144 |
| hsa_circRNA_0030122 | *VWA8* | 3-4 | F: 5'-ACATTGATCAGTTTCAGACTCTCT-3'<br>R: 5'-AAACATCTTGCCCCAAAAGATCC-3' | 55 | 89 |
| **Linear RNAs** | | | | | |
| | *BMPR2* | / | F: 5'-GCAGCAGTATACAGATAGGTGAG-3'<br>R: 5'-AGCAAGTCTTTGTTGCAGGG-3' | 60 | 398 |
| | *BACH1* | / | F: 5'-CTCTCGCTTCAGTCAGTCGG-3'<br>R: 5'-CGCTGTCCCTCCACAAAGAT-3' | 60 | 233 |
| | *PVT1* | / | F: 5'-CTTCCAGTGGATTTCCTTGC-3'<br>R: 5'-CATCTTGAGGGGCATCTTTT-3' | 60 | 125 |
| | *CCSER2* | / | F: 5'-GTGGAGTGTGACAATATGAAC-3'<br>R: 5'-GTGGCTGAGGTGGTAATG-3' | 63 | 123 |
| | *ATXN3* | / | F: 5'-TGCTCAACATTGCCTGAA-3'<br>R: 5'-ACTCCTCCTTCTGCCATT-3' | 63 | 117 |
| | *SIRT3* | / | F: 5'-CAAGGAGCTGTACCCTGGAAA-3'<br>R: 5'-CGACACTCTCTCAAGCCCATC-3' | 60 | 124 |

**Housekeeping gene**

| | | | | |
|---|---|---|---|---|
| *GAPDH* | For qPCR | F: 5'-CACTAGGCGCTCACTGTTCTC-3'<br>R: 5'-GACTCCACGACGTACTCAGC-3' | 59 | 350 |
| *GAPDH* | Face-to-face | F: 5'-GAAGGTGAAGGTCGGAGTC-3'<br>R: 5'-GAAGATGGTGATGGGATTTC-3' | 60 | 226 |
| *GAPDH* | Back-to-back | F: 5'-GCTGAGTACGTCGTGGAGTC-3'<br>R: 5'-GAGAACAGTGAGCGCCTAGTG-3' | 60 | / |

AT: Annealing Temperature. *, circRNA that contains parts of the introns.

**Table S7. Prediction of circRNA-miRNA binding sites.**

| circRNA | miRNA | Local AU | Matching type | Position |
|---|---|---|---|---|
| hsa_circ_0003169 | hsa-miR-130b-5p | GAAAGAG | 7mer-m8 | 182-201 |
| | hsa-miR-4682 | AACTCAGA | 8mer | 12-32 |
| hsa_circ_0024246 | hsa-miR-1301-3p | AGCTGCA | 7mer-m8 | 23-46 |
| | | AGCTGCA | 7mer-m8 | 229-254 |
| | hsa-miR-4660 | GAGCTGCA | 8mer | 25-45 |
| | | GAGCTGCA | 8mer | 229-253 |
| hsa_circ_0030122 | hsa-miR-623 | CAAGGGA | 7mer-m8 | 152-176 |
| | hsa-miR-7158-3p | TAGTTCA | 7mer-m8 | 12-32 |
| hsa_circ_0061363 | hsa-miR-29a-3p | TGGTGCT | 7mer-m8 | 143-165 |
| | hsa-miR-29b-3p | TGGTGCT | 7mer-m8 | 142-165 |
| | hsa-miR-29c-3p | TGGTGCT | 7mer-m8 | 143-165 |

Notes: 7mer-m8: an exact match to positions 2-8 of the mature miRNA (the seed + position 8); 8mer: an exact match to positions 2-8 of the mature miRNA (the seed + position 8) followed by an 'A'.
